# Carbon dioxide angiography-guided balloon pulmonary angioplasty for inoperable chronic thromboembolic pulmonary hypertension with iodinated contrast allergy

**DOI:** 10.1007/s12928-026-01261-7

**Published:** 2026-03-21

**Authors:** Ryo Takano, Jin Ueda, Hiroki Horinouchi, Tetsuya Fukuda, Takeshi Ogo

**Affiliations:** 1 Division of Pulmonary Circulation, Department of Cardiovascular Medicine, National Cerebral and Cardiovascular Center, Suita, Osaka Japan; 2 Department of Radiology, National Cerebral and Cardiovascular Center, Suita-si, Japan

A 61-year-old woman was referred for chronic thromboembolic pulmonary hypertension (CTEPH) and World Health Organization Functional Class (WHO-FC) III exertional dyspnea. She had a history of anaphylaxis to iodinated contrast agents. Right heart catheterization revealed a mean pulmonary arterial pressure (mPAP) of 49 mmHg and pulmonary vascular resistance (PVR) of 13.0 Wood units. Ventilation–perfusion scintigraphy revealed segmental perfusion defects (Fig. 1A), and gadolinium contrast-enhanced magnetic resonance angiography (MRA) identified peripheral pulmonary artery thromboembolic stenosis (Fig. 1B). The patient was diagnosed with distal-type CTEPH. Balloon pulmonary angioplasty (BPA) was considered appropriate because of the presence of distal lesions. However, BPA with iodinated contrast medium is contraindicated in patients with severe allergy. Therefore, we planned BPA guided by carbon dioxide (CO₂) instead of iodinated contrast medium. Transthoracic echocardiography and MRA revealed no intracardiac right-to-left shunt or pulmonary arteriovenous malformations, suggesting that CO₂ angiography was acceptable. Following selective insertion of a 6-Fr guiding catheter into the target vessels, digital subtraction angiography with breath-holding was performed using 20–40 mL of manually administered CO₂ injected over 1–2 s, with over 3 min intervals between injections. Subsequently, CO₂ angiography-guided BPA was performed (Fig. 1C–F, Video 1–4). Minor hemoptysis occurred during the second of eight procedures, but no serious complications occurred. Three months after the final BPA, her symptoms improved to WHO-FC I, with hemodynamic improvement, including reductions in mPAP and PVR to 21 mmHg and 3.6 Wood units, respectively. MRA demonstrated improvement in pulmonary artery stenosis (Fig. 1G).

**Fig. 1:**
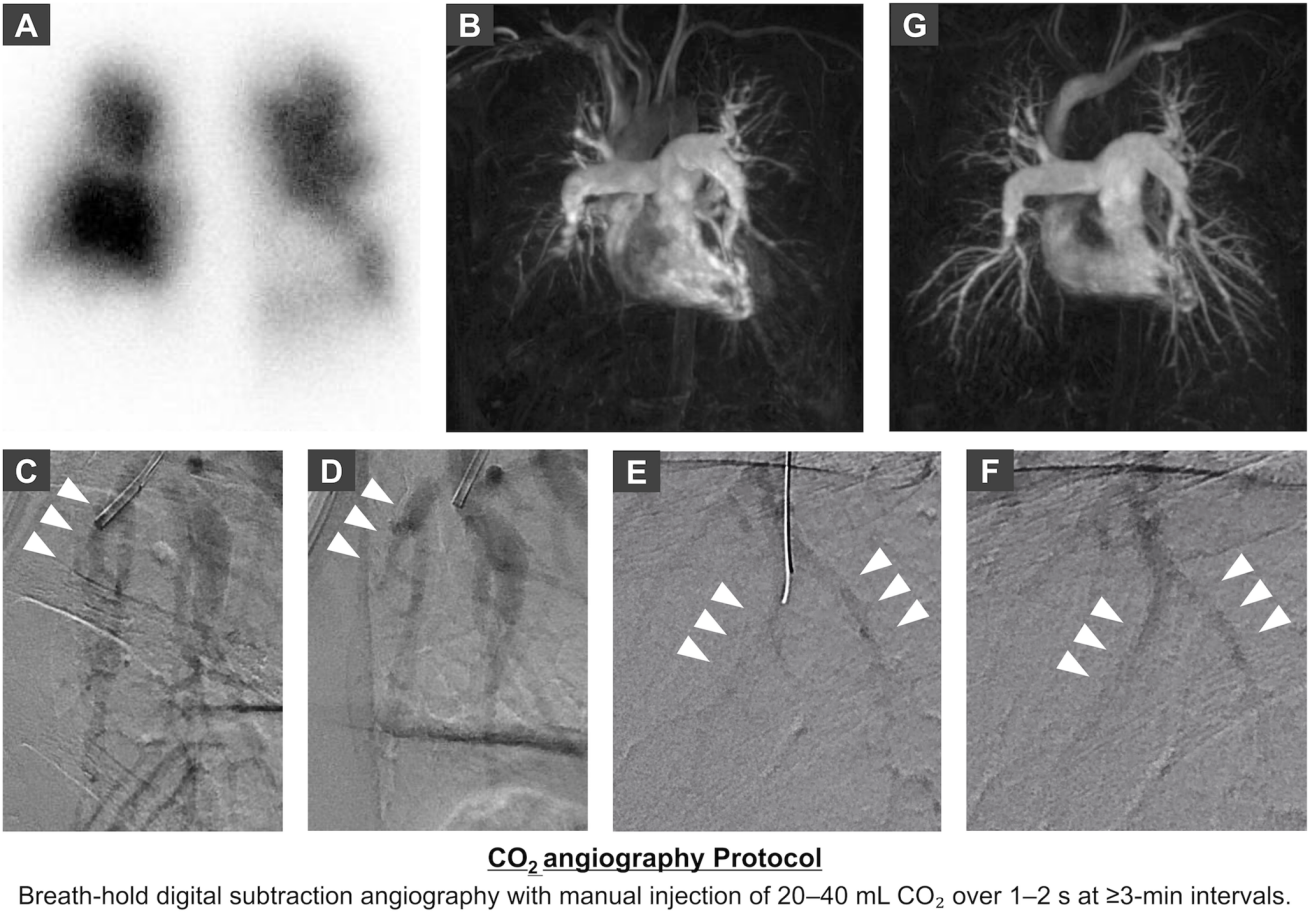
A Ventilation–perfusion scintigraphy. B, G Contrast-enhanced MRA (pre- and post-BPA). C–F CO₂ angiography of Lt.A10 (pre- and post-BPA) and Rt.A10 (pre- and post-BPA)

To our knowledge, this is the first case report of CO₂ angiography-guided BPA, which led to significant improvements in symptoms and hemodynamics. BPA is a catheter-based intervention using iodinated contrast. An alternative contrast medium is necessary for BPA in cases of anaphylaxis to iodinated contrast medium. We previously reported BPA using gadolinium-based contrast agents [1]. Gadolinium is expensive and deposits in various organs, even in patients with preserved renal function [2]. Therefore, CO₂ angiography was chosen for BPA in this case because multiple procedures were necessary. CO₂ is inexpensive and has been safely used as an alternative contrast agent in endovascular procedures [3]. Because CO₂ angiography carries a risk of gas trapping in the pulmonary arteries, it was performed with careful hemodynamic monitoring and the capability to aspirate gas, if needed. No gas-related complications were observed during the procedure. Additionally, CO₂ angiography is prone to respiratory variability, resulting in poorer visualization compared to iodinated contrast agents, and may increase the risk of wire perforation and improper balloon sizing. To address these limitations, intravascular ultrasound was used for lesions in which CO₂ assessment was insufficient, although most lesions were sized based on CO₂ angiography. BPA was effective without serious complications. In conclusion, CO₂ angiography-guided BPA may be an alternative for patients with inoperable CTEPH and anaphylaxis to iodinated contrast agents.

## Supplementary Information

Below is the link to the electronic supplementary material.

Supplementary Material 1

Supplementary Material 2

Supplementary Material 3

Supplementary Material 4

## Data Availability

The underlying data are not publicly available owing to privacy and ethical considerations.
